# Genomic characterization of *Klebsiella pneumoniae* carbapenemase-producing *Klebsiella pneumoniae* (KPC-Kp) strains circulating in three university hospitals in Northern Italy over three years

**DOI:** 10.1186/s13756-024-01429-x

**Published:** 2024-07-03

**Authors:** Valeria Fox, Davide Mangioni, Silvia Renica, Agnese Comelli, Antonio Teri, Michela Zatelli, Beatrice Silvia Orena, Cristina Scuderi, Annalisa Cavallero, Marianna Rossi, Maddalena Casana, Ludovica Mela, Alessandra Bielli, Rossana Scutari, Paola Morelli, Lisa Cariani, Erminia Casari, Chiara Silvia Vismara, Caterina Matinato, Annapaola Callegaro, Barbara Bottazzi, Barbara Cassani, Carlo Federico Perno, Andrea Gori, Antonio Muscatello, Alessandra Bandera, Claudia Alteri

**Affiliations:** 1 Department of Oncology and Hemato-Oncology, University of Milan, Milan, Italy; 2 Infectious Diseases Unit, Fondazione IRCCS Ca’ Granda Ospedale Maggiore Policlinico, Milan, Italy; 3 Department of Pathophysiology and Transplantation, University of Milan, Milan, Italy; 4 Microbiology Unit, Fondazione IRCCS Ca’ Granda Ospedale Maggiore Policlinico, Milan, Italy; 5 Residency in Microbiology and Virology, Università degli Studi di Milano, Milan, Italy; 6 Microbiology Unit, Humanitas Clinical and Research Center IRCCS, Rozzano, Milan, Italy; 7 Microbiology Unit, Fondazione IRCCS San Gerardo dei Tintori, Monza, Italy; 8 Infectious Diseases Unit, Fondazione IRCCS San Gerardo dei Tintori, Monza, Italy; 9 Infectious Diseases Unit, Humanitas Clinical and Research Center IRCCS, Rozzano, Milan, Italy; 10 Complex Unit of Clinical Microbiology, ASST Grande Ospedale Metropolitano Niguarda, Milan, Italy; 11 Humanitas Clinical and Research Center IRCCS, Rozzano, Milan, Italy; 12 Department of Medical Biotechnology and Translational Medicine, University of Milan, Milan, Italy; 13 Multimodal Research Area, Bambino Gesù Children Hospital IRCCS, Rome, Italy; 14 Division of Infectious Diseases, L. Sacco University Hospital, Milan, Italy

**Keywords:** *K. pneumoniae* carbapenemase-producing *Klebsiella pneumoniae* (KPC-Kp), WGS, Genomic epidemiology, Antimicrobial resistance (AMR)

## Abstract

**Objectives:**

Genomic surveillance of *Klebsiella pneumoniae* carbapenemase-producing *Klebsiella pneumoniae* (KPC-Kp) is crucial for virulence, drug-resistance monitoring, and outbreak containment.

**Methods:**

Genomic analysis on 87 KPC-Kp strains isolated from 3 Northern Italy hospitals in 2019-2021 was performed by whole genome sequencing (WGS), to characterize resistome, virulome, and mobilome, and to assess potential associations with phenotype resistance and clinical presentation. Maximum Likelihood and Minimum Spanning Trees were used to determine strain correlations and identify potential transmission clusters.

**Results:**

Overall, 15 different STs were found; the predominant ones included ST307 (35, 40.2%), ST512/1519 (15, 17.2%), ST20 (12, 13.8%), and ST101 (7, 8.1%). 33 (37.9%) KPC-Kp strains were noticed to be in five transmission clusters (median number of isolates in each cluster: 5 [3-10]), four of them characterized by intra-hospital transmission. All 87 strains harbored Tn*4401*a transposon, carrying *bla*_KPC-3_ (48, 55.2%), *bla*_KPC-2_ (38, 43.7%), and in one case (1.2%) *bla*_KPC-33,_ the latter gene conferred resistance to ceftazidime/avibactam (CZA). Thirty strains (34.5%) harbored porin mutations; of them, 7 (8.1%) carried multiple Tn*4401*a copies. These strains were characterized by significantly higher CZA minimum inhibitory concentration compared with strains with no porin mutations or single Tn*4401*a copy, respectively, even if they did not overcome the resistance breakpoint of 8 ug/mL. Median 2 (IQR:1-2) virulence factors per strain were detected. The lowest number was observed in ST20 compared to the other STs (*p*<0.001). While ST307 was associated with infection events, a trend associated with colonization events could be observed for ST20.

**Conclusions:**

Integration of genomic, resistance score, and clinical data allowed us to define a relative diversification of KPC-Kp in Northern Italy between 2019 and 2021, characterized by few large transmission chains and rare inter-hospital transmission. Our results also provided initial evidence of correlation between KPC-Kp genomic signatures and higher MIC levels to some antimicrobial agents or colonization/infection status, once again underlining WGS's importance in bacterial surveillance.

**Supplementary Information:**

The online version contains supplementary material available at 10.1186/s13756-024-01429-x.

## Introduction

*Klebsiella pneumoniae* (*Kp*) is a Gram-negative bacterium, a common commensal organism of the human gut, but also a major concern in healthcare settings [1]. It represents a leading cause of healthcare-associated infections (HAI) worldwide, including severe conditions such as bloodstream infections and sepsis/septic shock [2].

Carbapenems are β-lactam antibiotics regarded as the last line for the treatment of infections caused by multidrug-resistant gram-negative bacteria, including *K. pneumoniae* [3]. Nonetheless, this bacterium has acquired resistance to this class of antibiotics (Carbapenem-resistant *K. pneumoniae*, CR-Kp), becoming an increasing threat to public health due to the limited treatment options [4]. According to the last European Centre for Disease Prevention and Control (ECDC) report, CR-Kp has been detected in all European countries, with the highest proportion in southern and eastern Europe [5, 6].

One of the major mechanisms of carbapenem resistance in *K. pneumoniae* is the production of *K. pneumoniae* carbapenemase (KPC), an enzyme able to hydrolyze carbapenems, making them ineffective [7]. KPC-producing *K. pneumoniae* (KPC-Kp) strains have been frequently associated with outbreaks and infections with high mortality rates, particularly in healthcare settings [8, 9].

In Italy, even though measures are being implemented to control the spread of KPC-Kp, the high prevalence in some areas and the emergence of new resistance mechanisms continue to pose a significant threat to public health [10, 11]. Therefore, it is crucial to investigate the epidemiology of KPC-Kp on both regional and national/international basis, to identify the factors driving KPC-Kp spread and persistence and to develop effective control strategies.

In recent years, genomic epidemiology has emerged as a powerful tool to investigate the transmission and evolution of bacterial pathogens, including KPC-Kp [12–14].

In this study, we aimed to investigate the genetic diversity of KPC-Kp strains, collected from three Northern-Italian university hospitals between 2019-2021, by whole genome sequencing (WGS). Specifically, we sought to determine the extent of circulation of KPC-Kp strains by genomic epidemiology and to identify the genetic factors associated with resistance, virulence, and clinical presentation.

## Materials and methods

### Collection of bacterial isolates

Ninety-five KPC-Kp strains were selected from 137 KPC-Kp strains consecutively collected between January 2019 and December 2021 from three university hospitals in the Milan area in Lombardy, Northern Italy, i.e., Fondazione IRCCS San Gerardo Hospital (from now on referred to as Hospital S), Fondazione IRCCS Ca’ Granda Ospedale Maggiore Policlinico di Milano (Hospital P), IRCCS Humanitas Research Hospital (Hospital H).

This study is part of a prospective collection of biological samples that aims to evaluate/characterize bacterial factors, gut microbiome, and immunological responses of consecutively enrolled patients with KPC-Kp colonization or infection in the same period in the three hospitals. Colonization or infection events were defined according to clinical characteristics reported in Supplementary Information.

Antimicrobial susceptibility to Meropenem (MEM), Ceftazidime/avibactam (CZA), Trimethoprim/sulfamethoxazole (STX), and Colistin (Col) was tested by broth microdilution using the Microscan WalkAway instrument (Beckman Coulter, Brea, United States) and the Thermo Scientific Sensititre plate (Thermo Fisher Scientific, Massachusetts, United States). Carbapenemase genes were detected by NXpert Carba-R (Cepheid, Sunnyvale, United States) [15].

### DNA extraction and Whole genome sequencing analysis

DNA extraction and whole genome sequence analysis, comprising read pre-processing and bacterial typing, are reported in the Supplementary Information.

### Phylogenetic analysis

Reference genomes for the most represented sequence types to be included in the phylogenetic analysis are listed in Supplementary Table 1 and were selected according to the criteria included in the Supplementary information.

A core single nucleotide polymorphisms (SNP) alignment was obtained with Snippy (v4.6.0) [16] using the ST307 *K. pneumoniae* KP47 (GenBank accession number OX359175) as reference, after removing regions predicted as possible recombinogenic regions by Gubbins (v3.2.1) [17] from all strains including the reference genomes, and used to perform a phylogenetic analysis by Maximum Likelihood (ML) method, as detailed in the Supplementary Information.

To better characterize the most prevalent STs, 5 different core SNP alignments were obtained similarly for each ST by aligning the isolate reads to the respective ST reference and were analyzed for the presence of potential transmission clusters, following the methods reported in the Supplementary information.

### Statistical analyses

Descriptive statistics are expressed as median values and interquartile range (IQR) for continuous data and number (percentage) for categorical data. Significant differences were assessed by the Fisher exact test or Kruskal–Wallis tests for categorical and continuous variables, respectively. All statistical analyses were performed SPSS software package for Windows (version 23.0, SPSS Inc., Chicago, IL). A *p*-value <0.05 was considered statistically significant.

Likelihood Ratio Test, followed by a multinomial logistic regression model to estimate 95% confidence intervals of odds ratios, was used to compare demographic and clinical characteristics between the overall study population and the samples selected for WGS analysis.

### Ethical and regulatory aspects

The study was registered by the Institutional Review Board of the coordinating Hospital Comitato Etico Brianza (#2555_09.08.2017) and was conducted following the standards of the Helsinki Declaration. Written informed consent was obtained from each study participant in all the participating Hospitals.

## Results

### Patients’ characteristics

Between January 2019 and December 2021, 136 KPC-Kp strains were consecutively collected in the 3 university hospitals. Ninety-four strains were selected for WGS, according to the number of KPC-Kp per hospital. The comparison of demographic and clinical characteristics of the selected samples with the general population affected by KPC-Kp strains was shown in Supplementary Table 2 and Supplementary Information.

After sequencing, one strain was excluded due to the absence of a *bla*_KPC_ gene, while two other strains were excluded due to suboptimal assembly quality. For 6 patients, two strains were collected, one coming from the colonization event, and one recovered upon the emergence of an infection. In the 4 cases where the infection events were found to be caused by the same strain of the colonization event (median [IQR] 33 [22.5-56.3] days between colonization and infection events isolation), we decided to keep only the strain coming from the first event (i.e., colonization). Thus, a total of 87 KPC-Kp strains, coming from 85 patients, were included in the final analysis (Supplementary Figure 1). More than half of the samples were collected in Hospital P (54/87, 62.1%), followed by Hospital H (24/87, 27.6%) and Hospital S (9/87, 10.3%).

The demographic and clinical characteristics of the 87 events finally included in the analysis are reported in Table 1. The median (IQR) age was 70.0 (60.5-78.5) years, with more than half of the population being male (58/87, 66.7%). Due to logistics limitations caused by the COVID-19 pandemic, most samples were retrieved between July-December 2019 (21/87, 24.2%) and January-June 2021 (23/87, 26.4%). More than two third of the samples were from colonization events (57/87, 65.5%) and the main isolation sites were rectal swabs (38/87, 43.7%), followed by urine (21/87, 24.1%), blood (15/87, 17.2%), abdomen (6/87, 6.9%) and other sites (7/87, 8.1%). As expected, the rectal swab was the isolation site associated with colonization samples (p<0.001), whereas blood was the one associated with infection samples (*p*<0.001).

**Table 1:** Demographic and samples’ characteristics of the study population composed by 87 KPC-Kp strains

**Characteristics**	**Overall (*N*=87)**	**Colonized (*N*=57)**	**Infected (*N*=30)**	***p*-value**^**a**^
**Demographics**				
Age, median (IQR)	70.0 (60.5, 78.5)	68.0 (60.0, 76.0)	76.5 (64.8, 81.8)	**0.023**
Sex, male	58 (66.7)	36 (63.2)	22 (73.3)	0.929
**Date of isolation**				
January – June 2019	15 (17.2)	15 (26.3)	0 (0)	**0.005**
July – December 2019	21 (24.2)	17 (29.8)	4 (13.4)	0.137
January – June 2020	4 (4.6)	3 (5.3)	1 (3.3)	0.690
July – December 2020	10 (11.5)	3 (5.3)	7 (23.3)	**0.018**
January – June 2021	23 (26.4)	15 (26.3)	8 (26.7)	0.976
July – October 2021	14 (16.1)	4 (7.0)	10 (33.3)	**0.004**
**Isolation site**				
Rectal swab	38 (43.7)	38 (66.7)	0 (0)	**<0.001**
Urine	21 (24.1)	11 (19.3)	10 (33.3)	0.331
Blood	15 (17.2)	0 (0)	15 (50.0)	**<0.001**
Abdomen-pelvis	6 (6.9)	2 (3.5)	4 (13.4)	0.138
Other^b^	7 (8.1)	6 (10.5)	1 (3.3)	0.207
**Hospital**				
P	54 (62.1)	41 (71.9)	13 (43.3)	**0.043**
H	24 (27.6)	11 (19.3)	13 (43.3)	0.108
S	9 (10.3)	5 (8.8)	4 (13.4)	0.530

Data are expressed as median (IQR), or N (%)

^a^Two-sided *P*-values were calculated by Mann-Whitney test, or Chi-square test, as appropriate

^b^Others include: Other surveillance swabs (*n*=4) and Respiratory samples (*n*=3)

### Whole genome sequencing statistics

Whole genome assemblies of the KPC-Kp strains displayed a median (IQR) number of contigs of 83 (71 - 114), with a median (IQR) *N*_50_ of 174 881 (151 303 – 200 863) bp, while the strains’ total genome sizes ranged from 5.24 to 6.10 Mb.

### Prevalence of ST, K, O loci and resistance to β-lactam/ β-lactamase inhibitors

**Fig. 1:**
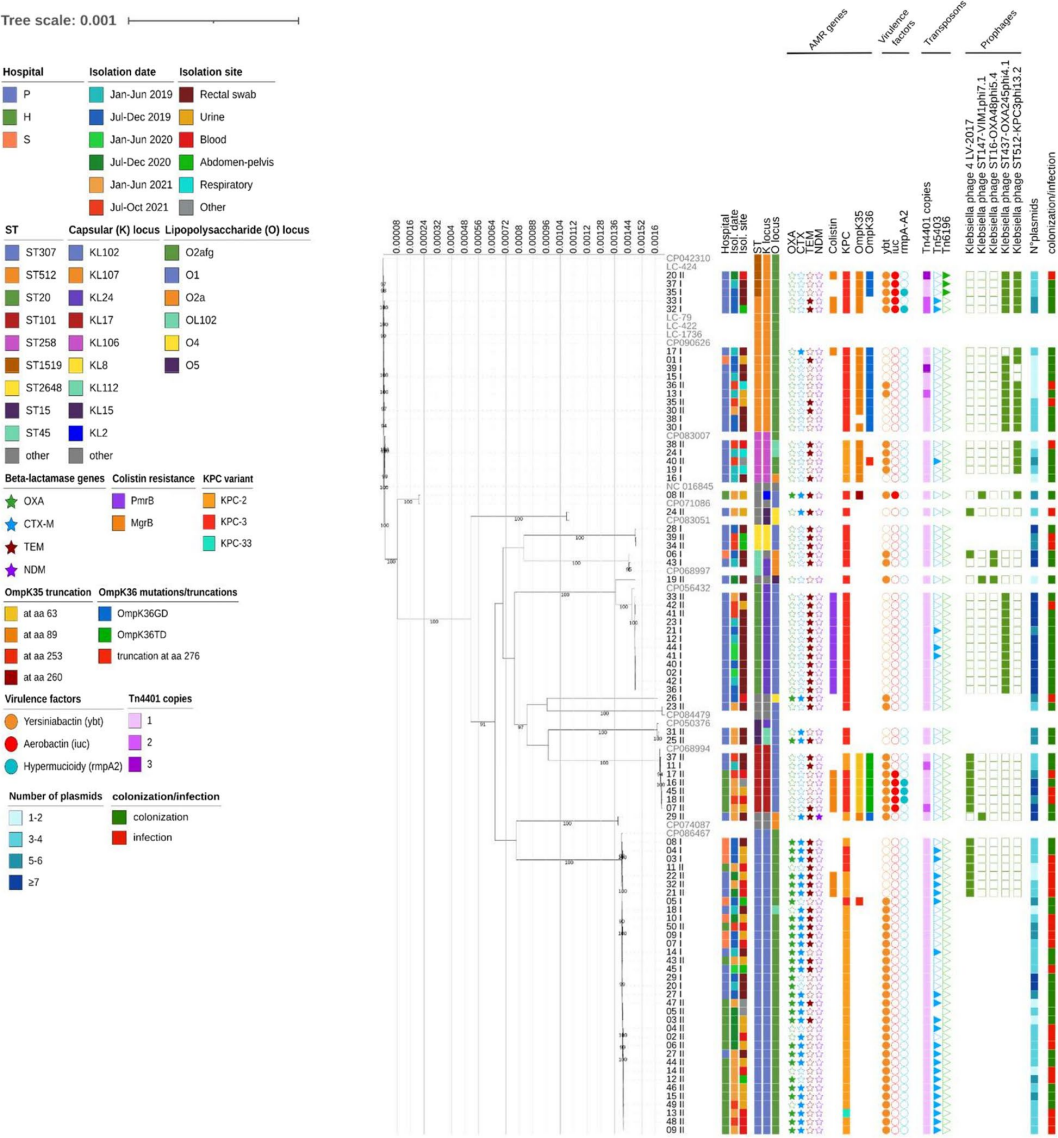
Estimated Maximum Likelihood phylogenetic analysis of KPC-Kp (*N*=87) isolated from three hospitals in Northern Italy and reference genomes (*N*=16). The Maximum Likelihood tree was inferred from a core-SNP alignment of 25,322 bp. The phylogeny was estimated with IqTree using the best-fit model of nucleotide substitution GTR+F+G4 with 1,000 replicates and fast bootstrapping. The numbers on the leaves represent the sample IDs. Reference genomes are displayed in grey. Bootstrap values higher than 90 are displayed on branches. Information regarding samples is annotated: hospital of origin, isolation date, isolation site, sequence type (ST), capsular locus (K locus), lipooligosaccharide locus (O locus), and the presence (solid figures) or absence of antimicrobial resistance genes, virulence factors and transposons, number of plasmids and bacteriophages, colonization, or infection status

The Maximum Likelihood (ML) tree constructed on a coreSNP alignment of 25,322 bp revealed the clustering of the strains based on the multilocus sequence type (ST) (Fig. 1). Overall, 15 different STs were found; the predominant ones were ST307 (35/87, 40.2%), ST512/1519, sharing 1 allele difference and clustering together in the ML tree (15/87, 17.2%), ST20 (12/87, 13.8%), ST101 (7/87, 8.1%), and ST258 (5/87, 5.8%), while remaining isolates (13/87, 14.9%) belonged to STs represented by less than 5 isolates.

The most prevalent capsular (K) locus identified was KL102 (35/87, 40.2%), followed by KL107 (15/87, 17.2%), KL24 (13/87, 14.9%), KL17 (7/87, 8.1%), KL106 (5/87, 5.8%), while other capsular loci were found in less than 5 isolates (12/87, 13.8%). Among the O loci, the most common was the O2afg (51/87, 58.6%), followed by O1 (26/87, 29.9%), O2a (4/87, 4.6%), OL102 (3/87, 3.5%), O4 (2/87, 2.3%), and O5, found in only one strain (1.2%).

As known, STs, K loci, and O loci were found to be closely associated with each other (Supplementary Figure 2, Panels A and B).

Most of the 87 samples carried *bla*_KPC-3_ (48/87, 55.2%), followed by *bla*_KPC-2_ (38/87, 43.7%) and the ceftazidime/avibactam (CZA)-conferring resistance *bla*_KPC-33_ (1/87, 1.1%). The *bla*_KPC_ genes were always carried by transposon Tn*4401*, always of isoform a (Tn*4401*a), but localized in different insertion sites (Supplementary Figure 3). Thirty strains (34.5%) also harbored mutations in OmpK35 and OmpK36 proteins, already associated with reduced susceptibility to CZA and MEM [18, 19]*.*

### *Genetic divergence, transmission clusters, and *β*-lactam/ β-lactamase inhibitors resistance against STs*

The predominant 5 STs were then in-depth characterized, by looking at the Minimum Spanning Trees (MSTs) and the SNP distances.

**Fig. 2:**
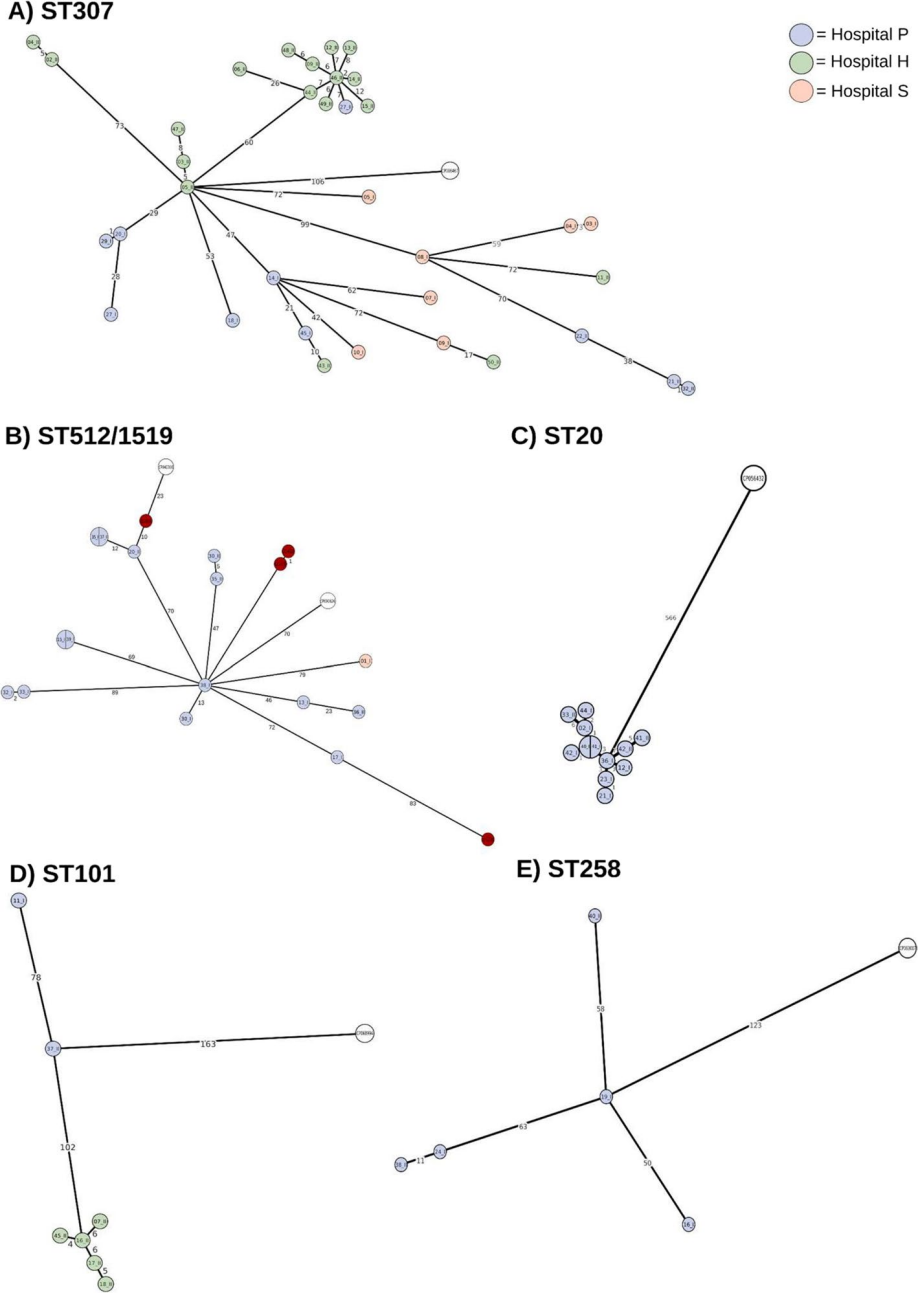
Minimum Spanning Trees (MSTs) of the pairwise SNP distance of strains within the 5 predominant STs. Branches display the pairwise SNP distances, while nodes report the strains ID and are colored based on the hospital of origin, blue= P, green=H, salmon=S. Strains colored in red are strains described in a recent Italian outbreak [22]. A) ST307, constructed on a coreSNP of 773 bp; B) ST512/1519, constructed on a coreSNP of 489 bp; C) ST20, constructed on a coreSNP of 587 bp; D ST101, constructed on a coreSNP of 271 bp; E) ST258, constructed on a coreSNP of 229 bp

The 35 strains belonging to ST307 had a median (IQR) SNP distance of 105 (72-150) and were distributed in all three Hospitals. Most of the 35 strains harbored the *bla*_KPC-2_ (30/35, 85.7%), while the remaining 5 strains harbored the *bla*_KPC-3_ (4/35, 11.4%) and *bla*_KPC-33_ (1/35, 2.9%). The Tn*4401*a flanking sequences detected were ATTGA/ATTGA, corresponding to a single insertion site in the epidemic IncFII(K) pKpQIL plasmid (Supplementary Figure 3). Regarding mutations in the porins, only one strain harboring *bla*_KPC-3_ also carried a truncation at amino acid (aa) 253 of OmpK35. The 35 ST307 strains were also characterized by the presence of 2 transmission clusters. The first cluster comprised only 3 strains harboring the *bla*_KPC-2_ and was characterized by a median (min-max) SNP distance of 8 (5-11). These 3 strains were all isolated from Hospital H between July 2020 and May 2021, coming in two cases from the neurological rehabilitation ward (05_II and 47_II) and in one case from the hepatology ward (03_II). The other cluster involved 10 strains, harboring the *bla*_KPC-2_ in 9 cases and *bla*_KPC-33_ in one case, and isolated from February to October 2021 at Hospital H (isolated from 7 different wards), out of one isolated at Hospital P. This cluster was characterized by a median (IQR) SNP distance of 11 (9-13) (Fig. 2A).

The 15 strains belonging to ST512/1519 were predominantly coming from Hospital P (14/15, 93.3%), with only 1 strain coming from Hospital S. All 15 strains harbored the *bla*_KPC-3_ (15/15, 100%). Six different 5 bp flanking sequences were found, corresponding to a single insertion site in 10 strains and multiple insertion sites in 5 strains (Supplementary Figure 3), implying for the latter the presence of multiple copies of Tn*4401*a inserted in different genome regions. Regarding mutations in the porin proteins, all the 15 ST512/1519 strains harbored a truncation at aa 89 of OmpK35. Thirteen out of 15 strains also carried a 2 aa insertion in position 134-135 of OmpK36 (OmpK36GD) [19]. The three ST1519 strains, all coming from Hospital P and isolated between January 2019 and September 2020 from the hepatological surgery unit (20_II and 37_I) and from the hematology ward (35_I), belonged to a transmission cluster characterized by a median (min-max) SNP distance of 12 (0-12) (Fig. 2B). No cluster was identified among the ST1512 strains.

The 12 strains belonging to ST20 were all isolated from Hospital P and were all part of a transmission cluster characterized by a low genetic divergence (median [IQR] pairwise SNP distance of 6 [4–9]) (Fig. 2C). Strains were isolated between 7^th^ January 2019 (ID 42_I) and 28^th^ August 2021 (ID 42_II), and all but one were related to colonization events. Similarly, to ST512/1519, all the 12 ST20 strains harbored the *bla*_KPC-3_ (12/12, 100%). The transposon displayed the 5 bp flanking sequences ATTGA/ATTGA in all the ST20 strains (100%) as a single insertion site (Supplementary Figure 3). No mutations in the OmpK proteins were detected.

The 7 strains belonging to ST101 had a median (IQR) pairwise SNP distance of 78 (7-105). Two of these strains (28.6%), both isolated from Hospital P, harbored the *bla*_KPC-2_ on a Tn*4401*a displaying in one case a single flanking sequence ATTGA/ATTGA, and in the other case multiple flanking sequences ATTGA/GTTTC-ATTGA/GTTTC (Supplementary Figure 3). The remaining 5 strains (71.4%), all from Hospital H, harbored the *bla*_KPC-3_, in one case on a Tn*4401*a inserted in multiple sites flanked by the ATTGA-ATTGA/TATCT sequences and in 4 cases on Tn*4401a* displaying the flanking sequences ATTGA/TATCT. (Supplementary Figure 3). These strains, isolated from the 29^th^ of January to the 6^th^ of September and coming from infection events in two cases and from colonization events in three cases, formed a transmission cluster characterized by a median (IQR) SNP distance of 7 (6-8) (Fig. 2D). All the seven ST101 strains carried alterations in porin proteins, like truncation at aa 63 of OmpK35 and GD insertion in position 134-135 of OmpK36 (OmpK36GD).

The 5 strains belonging to ST258 were all coming from Hospital P and showed a high diversification, with a median (IQR) pairwise SNP distance of 71(59-76) and no evidence of transmission clusters. All these strains harbored the *bla*_KPC-2_ (5/5, 100%) on Tn*4401*a displaying a single ATTGA/ATTGA flanking sequence (Supplementary Figure 3). Regarding alterations in the porin proteins, the 5 ST258 strains harbored the truncation at aa 89 of OmpK35 (truncation at aa 89).

Regarding the less-represented STs, 12/13 carried *bla*_KPC-3_ (92.3%). Of these strains, only one belonging to ST395 carried a truncated OmpK35 at aa 260. A single strain, belonging to ST147 (ID 29_II) carried *bla*_KPC-2_ together with *bla*_NDM-1_. This strain also displayed the combination of OmpK35 truncation at aa 89 and OmpK36GD insertion.

### Phenotypic and genotypic correlation of antimicrobial resistance and other resistance mechanisms

The antimicrobial susceptibility to MEM, CZA, STX, and Col was available for 71/87 KPC-Kp strains (Table 2).

**Table 2:** Antimicrobial resistance genomic signatures against phenotypic antimicrobial resistance profiles. In the 71 strains for which the Minimum inhibitory concentration (MIC) data for Meropenem (MEM), Ceftazidime/Avibactam (CZA), Colistin (CST), and Trimethoprim/Sulfamethoxazole (SXT) were available, differences in the phenotypic antimicrobial resistance were evaluated according to *bla*_KPC_ gene variants, colistin mutations, presence of genes associated with trimethoprim and sulfonamide resistance, number of Tn*4401*a copies and porin mutations. Only one strain (29_II, carrying both KPC-2 and NDM-1) was found resistant (MIC: 16 µg/ml) to the Meropenem/Vaborbactam (MEM/VAB) combination

**Mechanism**		**Meropenem**			**Ceftazidime/Avibactam**			**Colistin**			**Trimethoprim/sulfamethoxazole**		
		**(MEM)**			**(CZA)**			**(CST)**			**(SXT)**		
		MIC breakpoint for resistance >8 N(%)^a^	MIC Median (IQR)^b^	*p*-value^c^	MIC breakpoint for resistance >8 N(%)^a^	MIC Median (IQR)^b^	*p*-value^c^	MIC breakpoint for resistance >2 N(%)^a^	MIC Median (IQR)^b^	*p*-value^c^	MIC breakpoint for resistance >4 N(%)^a^	MIC Median (IQR)^b^	*p*-value^c^
***bla***_KPC_**gene**^d^	***bla***_KPC-2 _***N=29***	25 (86.2)	16 (16-32)	1) 0.368 2) 0.914	0 (0.0)	2 (1-2)	1) 1.000 2) 0.002	-	-	-	-	-	-
	***bla***_KPC-3 _***N=41***	31 (75.6)	16 (16-32)		1 (2.4)	2 (2-2)		-	-		-	-	
**Porin mutations**	**none *N=44***	31 (70.5)	16 (8-16)	1) 0.036 2) <0.001	1 (2.3)	2 (2-2)	1) 1.00 2) 0.002	-	-	-	-	-	-
	**At least one *N=27***	25 (92.6)	32 (32-32)		1 (3.7)	2 (2-4)		-	-		-	-	
**Tn*4401* copies**	**1 *N=65***	50 (76.9)	16 (16-32)	1) 0.331 2) 0.006	2 (3.1)	2 (2-2)	1) 1.000 2) 0.001	-	-	-	-	-	-
	**> 1 *N=6***	6 (100)	32 (32-32)		0 (0.0)	6 (4-8)		-	-		-	-	
**1 Tn*4401*copy and no porin mutations *N=44***		31 (70.5)	16 (8-16)	1) 0.026 2) <0.001	1 (2.3)	2 (2-2)	1) 0.938 2) <0.001	-	-	-	-	-	-
**1 Tn*4401*copy and at least 1 porin mutation *N*=21**		19 (90.5)	32 (32-32)		1 (4.8)	2 (2-2)		-	-		-	-	
**>1 Tn*4401*copy and at least 1 porin mutation *N*=6**		6 (100.0)	32 (32-32)		0 (0.0)	6 (4-8)		-	-		-	-	
**Colistin mutations**	**none *N*=51**	-	-	-	-	-	-	1 (2.0)	2 (0.5-2)	1) <0.001 2) 0.001	-	-	-
	**MgrB or PmrB *N*=20**	-	-		-	-		7 (35.0)	2 (2-4)		-	-	
**Trimethoprim and sulfonamide mutations**	**none *N*=21**	-	-	-	-	-	-	-	-	-	0 (0.0)	2 (2-4)	1) 0.027 2) <0.001
	***dfr*A or *sul*** *N=50*	-	-		-	-		-	-		11 (22.0)	4 (4-4)	

^a^Clinical Minimum Inhibitory Concentration (MIC) breakpoint for resistance according to “The European Committee on Antimicrobial Susceptibility Testing. Breakpoint tables for interpretation of MICs and zone diameters. Version 14.0, 2024. http://www.eucast.org."

^b^Median and interquartile range (IQR) of MIC values (in µg/ml) for each of the antibiotics reported

^c^Two-sided *p*-values were calculated by 1) Fisher exact test and Chi-square for trend, for comparison of categorical variables among two and three independent groups, respectively, and 2) Kruskal–Wallis and Mann-Whitney tests for comparison of continuous variables among two or three groups, respectively

^d^*bla*_KPC-33_ (*N*=1) is not included

First, we investigated the role of *bla*_KPC_, alterations in porins, and multiple copies of the *bla*_KPC_-harbouring transposon in affecting the efficacy of the novel β-lactam/ β-lactamase inhibitors CZA and MEM, as previously proven [18–22].

Even if no difference was found in the number of strains that overcame the clinical resistance breakpoint for CZA, a significantly higher CZA MIC was associated with strains carrying the *bla*_KPC-3_ compared to those carrying the *bla*_KPC-2_ variant (median [IQR] MIC of 2 [2-2] vs 2 [1-2] ug/mL, *p*=0.002), with strains carrying porin mutations compared with those did not (median [IQR] MICs of 2 [2-4] vs 2 [2-2] ug/mL, *p*=0.002), and with strains carrying multiple copies of the *bla*_KPC_-harbouring transposon compared to those carrying a single Tn*4401*a copy (median [IQR] MICs of 6 [4-8] vs 2 [2-2] ug/mL, *p*=0.001) (Table 2).

Even if almost all strains overcame the clinical resistance breakpoint for MEM, significantly higher MEM MIC was observed in strains carrying porin mutations or multiple copies of the blaKPC-harbouring transposon (Table 2).

Among the other resistance mechanisms identified, truncations in either the MgrB or PmrB proteins, putatively associated with phenotypic Col resistance [23, 24], were found in 24 samples (27.6%). MgrB protein truncation was detected in 12 strains belonging to ST101 (*N*=5), ST512/1519 (N=4), and ST307 (*N*=3), while PmrB protein truncation was detected in the other 12 strains, all belonging to ST20. In line with previous findings [23, 24], a higher percentage of strains displaying MgrB and PmrB truncations overcame the clinical resistance breakpoint of 2 ug/mL compared to the strains that did not display these mutations (7 [35.0%] vs. 1 [2.0%], *p*<0.001; median [IQR] MIC of 2 [2-4] vs 2 [0.5-2] ug/mL, *p*=0.001) (Table 2).

Sixty strains carried also genes associated with trimethoprim (*dfr*A gene) and sulfamethoxazole resistance (*sul*1 and *sul*2). Specifically, 34 strains carried one between a *dfr*A or a *sul* gene, while 26 strains carried both types. Of note, higher MIC values were observed in strains carrying these genes compared to those that did not (median [IQR] MIC of 2 [2-4] vs 4 [4-4] ug/mL, *p*<0.001), even if only 11/50 strains carrying these genes exhibited a clinical resistance breakpoint for STX (Table 2).

Only the strain carrying both KPC-2 and NDM-1 (29_II) was found resistant to the Meropenem/Vaborbactam (MEM/VAB) combination (MIC=16 ug/mL).

### Virulence factors and MGEs

Among the virulence factors identified, three different types of siderophores were found, namely enterobactin (87/87, 100%), yersiniabactin (53/87, 60.9%), and aerobactin (11/87, 12.6%). Moreover, a truncated *rmp*A2, a gene normally associated with the hypermucoidy phenotype, was identified (5/87, 5.8%). The distribution of these virulence factors against STs is shown in Supplementary Figure 4. Yersiniabactin genes were found widespread among the different STs, on a self-transmissible integrative conjugative element (ICEKp), while aerobactin and truncated *rmp*A2, carried by the same plasmid (homologous to plasmid pKpvST147B, GenBank accession n° CP040726), were found almost exclusively in ST101 (*N*=5 and *N*=3, respectively) and ST512/1519 (*N*=5 and *N*=2, respectively). Looking at the number of virulence factors against STs, ST20 displayed a lower median (IQR) number per strain compared to the other STs (1[1-1] vs. 3 [2-4] of ST101 vs. 2 [2-2]of ST307 and ST258, vs 1 [1-3] of ST512/1519, *p*<0.001), as it carried no other virulence factor except for enterobactin.

By plasmid prediction, all KPC-Kp strains were found to carry at least 1 plasmid, with a median [IQR] number of plasmids per strain of 4 (3-6). ST20 and ST101 were found to carry the highest number of plasmids (7 [7-7] and 6 [4-7], respectively vs 4 [3-4] plasmids for ST307, 3 [2-3] for ST512/1519, and 3 [2-4] for ST258, *p*<0.001).

A total of 5 intact prophages could be identified in 66 strains (75.9%), namely Klebsiella phage ST437-OXA245phi4.1 (in 26/87 strains, GenBank acc. n° NC_049448.1), Klebsiella phage ST512-KPC3phi13.2 (in 18/87 strains, GenBank acc. n° NC_049452.1), Klebsiella phage 4 LV-2017 (in 16/87 strains, GenBank acc. n° NC_047818.1), Klebsiella phage ST16-OXA48phi5.4 (in 3/87 strains, GenBank acc. n° NC_049450.1), and Klebsiella phage ST147-VIM1phi7.1 (in 3/87 strains, GenBank acc. n° NC_049451.1) (Fig. 1).

No virulence factors were identified on the detected intact bacteriophages.

### Association of ST, transmission clusters, resistance, and MGE with colonization vs infection events

Finally, we evaluated the association of different genotypic traits with colonization and infection events (Table 3). Strains belonging to ST307 were found to be associated with infection events (*p*=0.011), whereas a trend could be observed between strains belonging to ST20 and colonization events (*p*=0.051). Due to the straight correlation between capsular loci and STs, similar associations with infection and colonization events could be observed for the capsular types. Of note, the trend of association between colonization events and ST20 became significant with KL24 (*p*=0.030), as this locus was carried by all ST20 strains and by an additional ST45 strain.

As a trend, strains carrying ≥7 plasmids were found to be negatively associated with infection events (*P*=0.071). No other significant associations were found.

## Discussion

The extensive genetic characterization of 87 KPC-Kp strains, isolated from patients across 3 university hospitals in Northern Italy between 2019 and 2021, highlighted a relative diversification of the strains, with a huge circulation of different STs. The most common *K. pneumoniae* ST found was ST307, followed by ST512/1519, ST20, ST101 and ST258. In Italy, *K. pneumoniae* epidemiological scenario has mainly been characterized by a prevalence of ST512, but with the emergence, in recent years, of other high-risk STs, including ST307, ST101, ST258 [25–29], and ST1519 found in some outbreaks [22, 30, 31]. Our study showed the introduction of the ST307, which is already globally distributed and has been found with other high-risk clones in some countries [32–36]. We also found a consistent proportion of KPC-Kp isolates belonging to ST20, a clone that is currently not reported as highly circulating in Italy, but which has been previously found responsible for SHV-5 and NDM-1 (no KPC) producing outbreaks in neonatal wards in China and Greece, respectively [37, 38]. Thus, we cannot exclude that the acquisition of the *bla*_KPC_ genes in this clone occurred via plasmids in the last years in Italy.

By investigating the genetic relatedness of the predominant STs among the three Hospitals, we found the presence of an overall high genetic diversity among KPC-Kp strains, even within the same hospital, confirming the circulation of multiple clones. 37.9% of KPC-Kp strains were involved in five transmission clusters, but only two of them could be defined as major transmission chains because composed of 10 and 12 strains, respectively. Four of them are within-hospital clusters, while only one cluster composed of ST307 strains showed an inter-hospital transmission due to a single strain. Overall, the low number of large transmission chains and the near absence of inter-hospital transmission could suggest the effective adherence to infection control practices within the three University Hospitals.

Molecular analysis of carbapenemase genes identified *bla*_KPC-3_ and *bla*_KPC-2_ as the most represented variants, concordantly with Italian epidemiology [39, 40]. As previously reported, ST307 and ST258 exclusively harbored *bla*_KPC-2_, while ST512/ST1519, ST20 and ST101 prevalently harbored *bla*_KPC-3_ [41]. Of note, we found one single KPC-Kp isolate (belonging to ST307) to carry *bla*_KPC-33_ gene, a *bla*_KPC-2_ variant showing a single base mutation at G532T, causing the D179Y amino acid mutation, which has previously been linked to high resistance to the novel anti-KPC-Kp antibiotic combination CZA [42]. All *bla*_KPC_ genes were found on transposon Tn*4401*a [43], itself harbored by plasmids, underlying the importance of mobile genetic elements in carbapenems resistance spread.

A good correlation was observed between genomic signatures and phenotypic resistance profile. As an example, 25/27 strains harboring at least one mutation either in the OmpK35 or OmpK36 proteins showed a MEM MIC >8 ug/mL. A small portion of these strains, all belonging to ST512/1519 (*n*=5) and ST101 (*n*=2) and all but one carrying *bla*_KPC-3_, harbored multiple copies of Tn*4401*a. These strains also displayed a median higher MIC to CZA (even though they did not overcome the clinical resistance breakpoint), consistently with what has been observed in previous reports [22, 44, 45] and confirming how the resistance phenotype is often given by the synergy of different mechanisms [19, 20]. Even if most of these identified strains were not part of transmission clusters, our findings warn about the ongoing circulation of ST512/1519 and ST101 clones frequently characterized by higher MIC to novel β-lactam/β-lactamase inhibitors in Italy, as well described in a recent Italian outbreak [22].

When we tried to correlate the identified genomic signatures with the colonization and infection status, we found that 60% of infection events were associated with ST307. Of note, this ST has been previously associated with higher mortality and longer hospital stays compared with other clones [34], probably due to its genetic features that may contribute to increased fitness and pathogenicity [46]. On the contrary, a high number of plasmids and ST20 were both moderately associated with colonization events. This is because most of the strains carrying ≥ 7 plasmids (*n*=20) were from colonization events (*n*=18) and belonged to the ST20 cluster (*n*=10). Thus, it is possible that in this case, specific plasmids characterizing the ST20 cluster might have conferred specific advantages during colonization events, allowing for increased survival and extensive circulation of specific strains in hospital environments [47, 48].

This study has some limitations. Firstly, not all *Kp*-KPC samples collected were subjected to WGS. However, when the characteristics of the general KPC-Kp-affected population were compared with those of the sampled population, no major differences were highlighted, suggesting a good representation of the selected samples.

We used short-read sequencing, which may have restricted our ability to accurately identify plasmids and detect multiple copies of the Tn*4401* transposon. In this regard, our findings revealed multiple copies of the transposon, particularly in strains belonging to ST512 and ST1519 lineages. Notably, these strains showed high similarities to those recently described by Di Pilato et al. [22] (Fig. 2), who identified duplicated KPC-carrying Tn*4401* transposons, employing both real-time PCR and long-read sequencing. Thus, our results appear to align with previous observations, suggesting consistency in our findings.

Finally, antimicrobial susceptibility testing results were not available for the whole strains’ collection, due to the absence of growth observed for 16 strains at the time of antimicrobial susceptibility testing.

Overall, through the integration of genome analysis with clinical and epidemiological data, our study allowed us to thoroughly investigate the genetic diversity of the KPC-Kp STs circulating in three different Hospitals in Northern Italy, to correlate genomic signatures with resistance score and infection or colonization status, and to confirm the ongoing circulation of clones characterized by MIC values close to the clinical resistance breakpoint to novel β-lactam/ β-lactamase inhibitors in Italy. These findings emphasize once more the relevant role played by the WGS in tracking bacterial spread and in monitoring the emergence and circulation of resistance and virulence mechanisms. These findings also reinforce the idea that a comprehensive genetic characterization could provide new and relevant data on the KPC-Kp genomic signatures correlated with different antimicrobial susceptibility and colonization or infection status.

### Supplementary Information

Additional file 1: Supplementary information. Supplementary materials and methods and results.

Additional file 2: Supplementary material 1. SNPs identified among strains of the predominant STs.

Additional file 3: Supplementary material 2. List of samples sequenced in this study and relative accession numbers on ENA.

Additional file 4: Supplementary Figure 1. Flow chart of the isolates’ inclusion process.

Additional file 5: Supplementary Figure 2. Distribution of K and O loci among the 5 prevalent STs. Percentage of prevalent STs displaying K (A) and O loci (B). *P*-values are calculated with the Chi-square test.

Additional file 6: Supplementary Figure 3. Schematic representation of the Tn*4401*a flanking regions among the different STs. Graphical representation of the Tn*4401*a flanking regions among the different STs. The presence of more than 2 sequences denotes the presence of multiple transposon copies, integrated in different regions.

Additional file 7: Supplementary Figure 4. Distribution of virulence factors among the 5 prevalent STs. Number of virulence factors carried by strains belonging to the different STs. 1 = only enterobactin, 2 = enterobactin + yersiniabactin, 3= enterobactin + yersiniabactin + aerobactin, 4 = enterobactin + yersianabactin+ aerobactin + *rmpA2*

Additional file 8: Supplementary Table 1. Reference genomes included in phylogenetic analysis.

Additional file 9: Supplementary Table 2. Demographic, and clinical findings of general KPC-Kp population and the 95 KPC-Kp population selected for WGS.

## Data Availability

The 87 *Klebsiella pneumoniae* sequence data obtained in this study are openly available on the European Nucleotide Archive (ENA) under the accession number PRJEB64702. The list of samples and relative accession numbers is available in Supplementary Material 2.
