# Supplementary material for: Genomic characterization of Klebsiella pneumoniae carbapenemase-producing Klebsiella pneumoniae (KPC-Kp) strains circulating in three university hospitals in Northern Italy over three years
Source: Antimicrob Resist Infect Control. 2024 Jul 3;13:70. doi: 10.1186/s13756-024-01429-x (PMC11223429; doi:10.1186/s13756-024-01429-x)
Supplement: Supplementary file 1 — Additional file 1: Supplementary information. Supplementary materials and methods and results. [file 13756_2024_1429_MOESM1_ESM.docx]

**Supplementary Information**

**Materials and methods**

*Definition of colonization and infection events*

Infections were defined by the presence of significant bacterial load associated with clinical manifestations within the infection window period (± 3 days from specimen collection) [1]

Isolates were classified as colonization when identified through surveillance swabs or other biological sample (e.g., urine, abdominal drain tube, wound swab) AND no adverse clinical signs or symptoms related to the bacterial presence was documented.

*DNA extraction and Whole genome sequencing analysis*

Genomic DNA was extracted from *K. pneumoniae* pure cultures using the DNeasy UltraClean Microbial Kit (QIAGEN, Hilden, Germany), following manufacturer’s instructions. Libraries for whole genome sequencing were generated using the Illumina DNA Library Prep Kit (Illumina Inc. San Diego, CA, USA) and sequenced on an Illumina sequencing platform (Illumina Inc. San Diego, CA, USA).

*Pre-processing and bacterial typing*

Raw reads were trimmed for adapters and filtered for quality (average quality > 20) with Fastp (v0.20.1) [2] and quality checked after trimming with FastQC (v0.11.9) [3]. Taxonomic classification was performed by Kraken (v1.0), to exclude contaminations [4].

A *de novo* genome assembly was performed using SPAdes Genome Assembler (v3.14.1) [5], using the ‘-careful’ option, and quality of the assemblies was evaluated by Quast (v5.1) [6]. Annotation of the assembled contigs was performed with Prokka (v1.14.6) [7] and isolate typing was performed with mlst tool (v2.11) [8,9]. Additional typing was obtained by looking at the variation in the composition and structure of capsular polysaccharide (K) and the outer core of lipooligosaccharide (O) by Kaptive (v0.7.3) [10,11]. Kleborate (v2.2.0) [12] was used to detect antimicrobial resistance determinants and virulence factors. MobileElementFinder tool (v1.0.3) [13] was used for mobile genetic elements identification. MOB-suite (v3.1.2) was used for plasmids prediction from draft genome assemblies and clustering of highly similar plasmids in similarity groups [14,15]. The presence of intact bacteriophages was investigated using geNomad [16] and manually curated by searching the identified sequences with the Microbial Nucleotide BLAST [17], using the bacteriophages database and a cut off of 90% percentage identity and 60% query coverage. The presence of Tn*4401* transposons, their type, and their flanking sequences were detected by TETyper (v1.1) [18].

In particular, the genetic context of the transposons was identified by specifying a length of 5bp for the flanking sequences. When more than one flanking sequence was detected, the presence of multiple transposon copies inserted in different genetic contexts was hypothesized.

*Phylogenetic analysis*

Reference genomes for the most represented sequence types to be included in the phylogenetic analysis were selected from PATRIC and NCBI databases [19, https://www.ncbi.nlm.nih.gov/] based on the following criteria: i) Genome status complete; ii) first searching for antimicrobial resistant strains; iii) isolation site preferably in Italy, otherwise in other European Countries; iv) collection year between 2017 (two years before the first KPC-Kp isolates in this study) and 2021; v) good genome quality.

A core single nucleotide polymorphisms (SNP) alignment was obtained with Snippy (v4.6.0) [20] from all strains including the reference genomes, by aligning all isolates and reference genomes to the ST307 *K. pneumoniae* KP47 genome (GenBank acc. n° OX359175, isolated in Italy in 2018). The coreSNP alignment obtained was then used to perform a phylogenetic analysis by Maximum Likelihood (ML) method with IQTREE (v2.0.6) [21], using the best nucleotide substitution model GTR+F+G4 inferred by ModelFinder [22] with 1000 bootstrap replicates, after removing regions predicted as possible recombinogenic regions by Gubbins (v3.2.1) [23]. Phylogenetic tree was visualized and annotated using iTOL (v6.5.7) [24].

To better characterize the most prevalent STs, 5 different core SNP alignments were obtained similarly by aligning the isolate reads to the respective ST. Minimum Spanning trees (MST) were also constructed on these core SNP alignments with Grapetree (v1.5.0) [25] and pairwise SNP distances were calculated using snp-dists tool (<https://github.com/tseemann/snp-dists>).

A bootstrap value of 100% by ML tree and a threshold of 20 SNPs by MST were considered suggestive of potential transmission chains, consistently with other already published articles on bacterial divergence [26-28].

**Results**

*Comparison between selected and general KPC-Kp strains*

In Supplementary Table 2, the characteristics of the 136 KPC-Kp strains consecutively collected between January 2019 and December 2021 in the 3 study hospitals were compared with the 94 selected samples. Likelihood Ratio Test, followed by a multinomial logistic regression model to estimate 95% confidence intervals of odds ratios, was used to compare demographic and clinical findings between general and selected KPC-Kp strains. Overall, the study population was representative of the general population, with few exceptions. Indeed, infection events were quite higher in samples collected for WGS with respect to the general KPC-Kp population (37.2% vs. 31.6%, p-value=0.039). Rectal swabs were quite lower in samples collected for WGS with respect to the general KPC-Kp population (43.6% vs. 50.0%, p-value=0.028). Isolation date intervals were largely comparable even though a different prevalence between selected samples and the general population was detected in July-December 2019 and July-December 2021 (26.6% vs 19.1%, p-value=0.009 and 14.9% vs. 24.3%, p-value=<0.001).

**References**

1. CDC. National Healthcare Safety Network (NHSN) Patient Safety Component Manual. January 2021
2. Chen S, Zhou Y, Chen Y, Gu J. fastp: an ultra-fast all-in-one FASTQ preprocessor. *Bioinformatics*. 2018;34(17):i884-i890. doi:10.1093/bioinformatics/bty560
3. Andrews S. FastQC: a quality control tool for high throughput sequence data. 2010. Available online at: <http://www.bioinformatics.babraham.ac.uk/projects/fastqc>
4. Wood DE, Salzberg SL. Kraken: ultrafast metagenomic sequence classification using exact alignments. *Genome Biol*. 2014;15(3):R46. Published 2014 Mar 3. doi:10.1186/gb-2014-15-3-r46
5. Bankevich A, Nurk S, Antipov D, et al. SPAdes: a new genome assembly algorithm and its applications to single-cell sequencing. *J Comput Biol*. 2012;19(5):455-477. doi:10.1089/cmb.2012.0021
6. Gurevich A, Saveliev V, Vyahhi N, Tesler G. QUAST: quality assessment tool for genome assemblies. *Bioinformatics*. 2013;29(8):1072-1075. doi:10.1093/bioinformatics/btt086
7. Seemann T. Prokka: rapid prokaryotic genome annotation. Bioinformatics. 2014;30(14):2068-2069
8. Jolley KA, Maiden MC. BIGSdb: Scalable analysis of bacterial genome variation at the population level. *BMC Bioinformatics*. 2010;11:595. Published 2010 Dec 10. doi:10.1186/1471-2105-11-595
9. Seemann T. mlst tool. Available at <https://github.com/tseemann/mlst>
10. Wyres KL, Wick RR, Gorrie C, et al. Identification of Klebsiella capsule synthesis loci from whole genome data. *Microb Genom*. 2016;2(12):e000102. Published 2016 Dec 12. doi:10.1099/mgen.0.000102
11. Lam MMC, Wick RR, Judd LM, Holt KE, Wyres KL. Kaptive 2.0: updated capsule and lipopolysaccharide locus typing for the *Klebsiella pneumoniae* species complex. *Microb Genom*. 2022;8(3):000800. doi:10.1099/mgen.0.000800
12. Lam MMC, Wick RR, Watts SC, Cerdeira LT, Wyres KL, Holt KE. A genomic surveillance framework and genotyping tool for *Klebsiella pneumoniae* and its related species complex. *Nat Commun.* 2021;12(1):4188. Published 2021 Jul 7. doi:10.1038/s41467-021-24448-3
13. Johansson MHK, Bortolaia V, Tansirichaiya S, Aarestrup FM, Roberts AP, Petersen TN. Detection of mobile genetic elements associated with antibiotic resistance in *Salmonella enterica* using a newly developed web tool: MobileElementFinder. *J Antimicrob Chemother*. 2021;76(1):101-109. doi:10.1093/jac/dkaa390
14. Robertson J, Nash JHE. MOB-suite: software tools for clustering, reconstruction and typing of plasmids from draft assemblies. *Microb Genom*. 2018;4(8):e000206. doi:10.1099/mgen.0.000206
15. Robertson J, Bessonov K, Schonfeld J, Nash JHE. Universal whole-sequence-based plasmid typing and its utility to prediction of host range and epidemiological surveillance. *Microb Genom*. 2020;6(10):mgen000435. doi:10.1099/mgen.0.000435
16. Camargo AP, Roux S, Schulz F, Babinski M, Xu Y, Hu B, Chain PSG, Nayfach S, & Kyrpides NC. 2023. Identification of mobile genetic elements with geNomad. Nature biotechnology, 10.1038/s41587-023-01953-y
17. available at <https://blast.ncbi.nlm.nih.gov/Blast.cgi?PAGE_TYPE=BlastSearch&BLAST_SPEC=MicrobialGenomes>
18. Sheppard AE, Stoesser N, German-Mesner I, et al. TETyper: a bioinformatic pipeline for classifying variation and genetic contexts of transposable elements from short-read whole-genome sequencing data. *Microb Genom*. 2018;4(12):e000232. doi:10.1099/mgen.0.000232
19. Wattam AR, Davis JJ, Assaf R, et al. Improvements to PATRIC, the all-bacterial Bioinformatics Database and Analysis Resource Center. *Nucleic Acids Res*. 2017;45(D1):D535-D542. doi:10.1093/nar/gkw1017
20. Seemann T (2015) Snippy: fast bacterial variant calling from NGS reads. Available online at: <https://github.com/tseemann/snippy>.
21. Nguyen LT, Schmidt HA, von Haeseler A, Minh BQ. IQ-TREE: a fast and effective stochastic algorithm for estimating maximum-likelihood phylogenies. *Mol Biol Evol*. 2015;32(1):268-274. doi:10.1093/molbev/msu300
22. Kalyaanamoorthy S, Minh BQ, Wong TKF, von Haeseler A, Jermiin LS. ModelFinder: fast model selection for accurate phylogenetic estimates. *Nat Methods*. 2017;14(6):587-589. doi:10.1038/nmeth.4285
23. Croucher NJ, Page AJ, Connor TR, Delaney AJ, Keane JA, Bentley SD, Parkhill J, & Harris SR. 2015. Rapid phylogenetic analysis of large samples of recombinant bacterial whole genome sequences using Gubbins. Nucleic acids research, 43(3), e15
24. Letunic I, Bork P. Interactive Tree Of Life (iTOL) v5: an online tool for phylogenetic tree display and annotation. *Nucleic Acids Res*. 2021;49(W1):W293-W296. doi:10.1093/nar/gkab301
25. Zhou Z, Alikhan NF, Sergeant MJ, et al. GrapeTree: visualization of core genomic relationships among 100,000 bacterial pathogens. *Genome Res*. 2018;28(9):1395-1404. doi:10.1101/gr.232397.117
26. David S, Reuter S, Harris SR, et al. Epidemic of carbapenem-resistant Klebsiella pneumoniae in Europe is driven by nosocomial spread. Nat Microbiol. 2019;4(11):1919-1929. doi:10.1038/s41564-019-0492-8
27. Liu X, Wang K, Chen J, et al. Clonal Spread of Carbapenem-Resistant Klebsiella pneumoniae Sequence Type 11 in Chinese Pediatric Patients. Microbiol Spectr. 2022;10(6):e0191922. doi:10.1128/spectrum.01919-22
28. Gorrie CL, Da Silva AG, Ingle DJ, et al. Key parameters for genomics-based real-time detection and tracking of multidrug-resistant bacteria: a systematic analysis. Lancet Microbe. 2021;2(11):e575-e583. doi:10.1016/S2666-5247(21)00149-X
