# Supplementary figures and images for: Genomic characterization of Klebsiella pneumoniae carbapenemase-producing Klebsiella pneumoniae (KPC-Kp) strains circulating in three university hospitals in Northern Italy over three years
Source: Antimicrob Resist Infect Control. 2024 Jul 3;13:70. doi: 10.1186/s13756-024-01429-x (PMC11223429; doi:10.1186/s13756-024-01429-x)

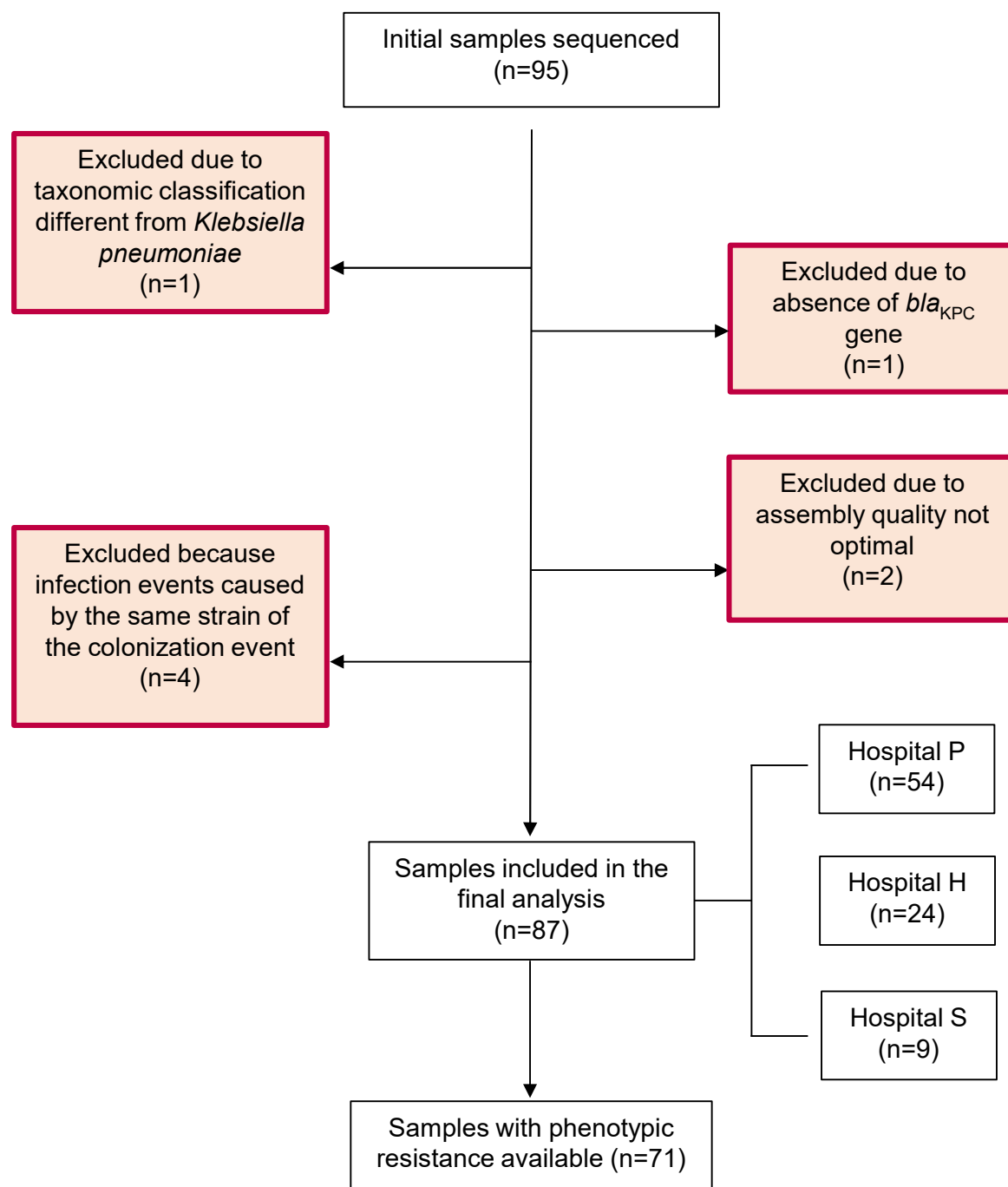

**Supplementary Figure 1.** Flow chart of the isolates' inclusion process.

Supplement: Supplementary file 4 — Additional file 4: Supplementary Figure 1. Flow chart of the isolates’ inclusion process. [file 13756_2024_1429_MOESM4_ESM.pdf]
