# Supplementary material for: Genomic characterization of Klebsiella pneumoniae carbapenemase-producing Klebsiella pneumoniae (KPC-Kp) strains circulating in three university hospitals in Northern Italy over three years
Source: Antimicrob Resist Infect Control. 2024 Jul 3;13:70. doi: 10.1186/s13756-024-01429-x (PMC11223429; doi:10.1186/s13756-024-01429-x)
Supplement: Supplementary file 5 — Additional file 5: Supplementary Figure 2. Distribution of K and O loci among the 5 prevalent STs. Percentage of prevalent STs displaying K (A) and O loci (B). P-values are calculated with the Chi-square test. [file 13756_2024_1429_MOESM5_ESM.pdf]

A)

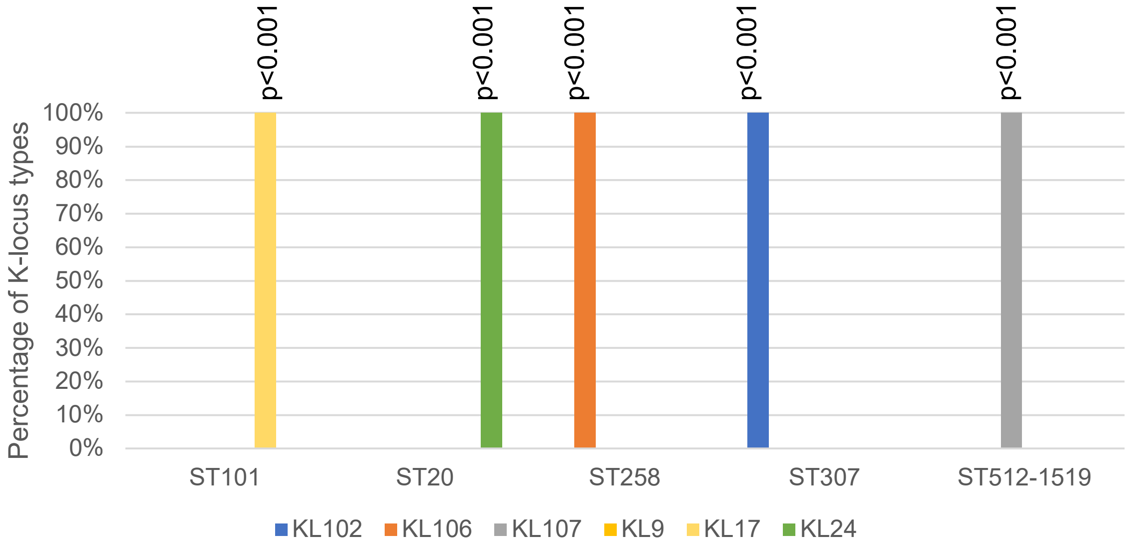


B)

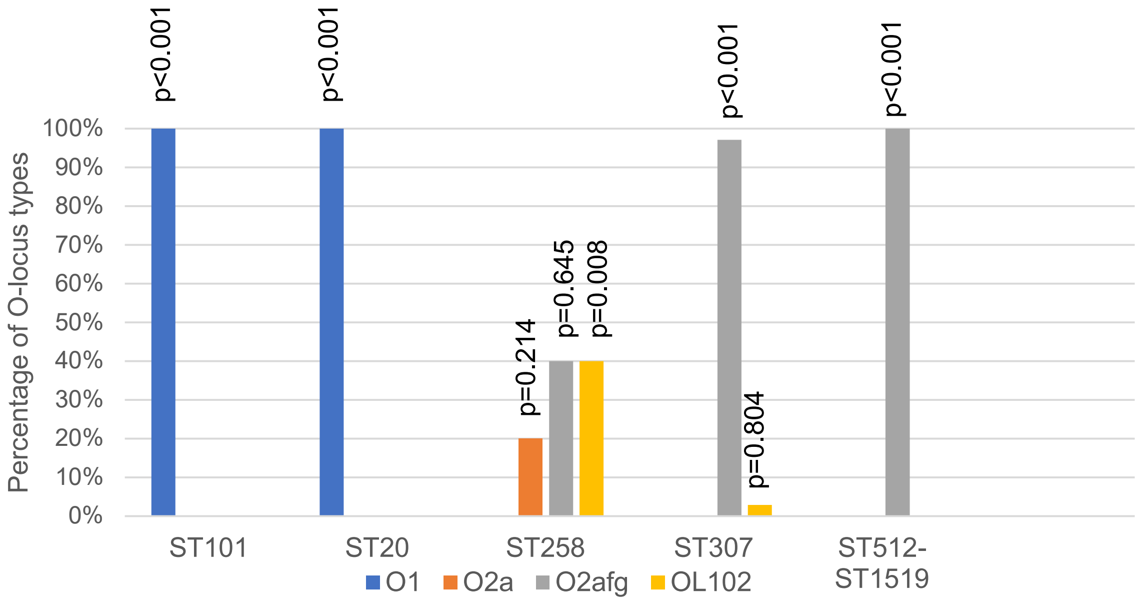


**Supplementary Figure 2**. **Distribution of K and O loci among the 5 prevalent STs**. Percentage of prevalent STs displaying K (A) and O loci (B). P-values are calculated with the Chi-square test.
