# Supplementary material for: Genomic characterization of Klebsiella pneumoniae carbapenemase-producing Klebsiella pneumoniae (KPC-Kp) strains circulating in three university hospitals in Northern Italy over three years
Source: Antimicrob Resist Infect Control. 2024 Jul 3;13:70. doi: 10.1186/s13756-024-01429-x (PMC11223429; doi:10.1186/s13756-024-01429-x)
Supplement: Supplementary file 6 — Additional file 6: Supplementary Figure 3. Schematic representation of the Tn4401a flanking regions among the different STs. Graphical representation of the Tn4401a flanking regions among the different STs. The presence of more than 2 sequences denotes the presence of multiple transposon copies, integrated in different regions. [file 13756_2024_1429_MOESM6_ESM.pdf]

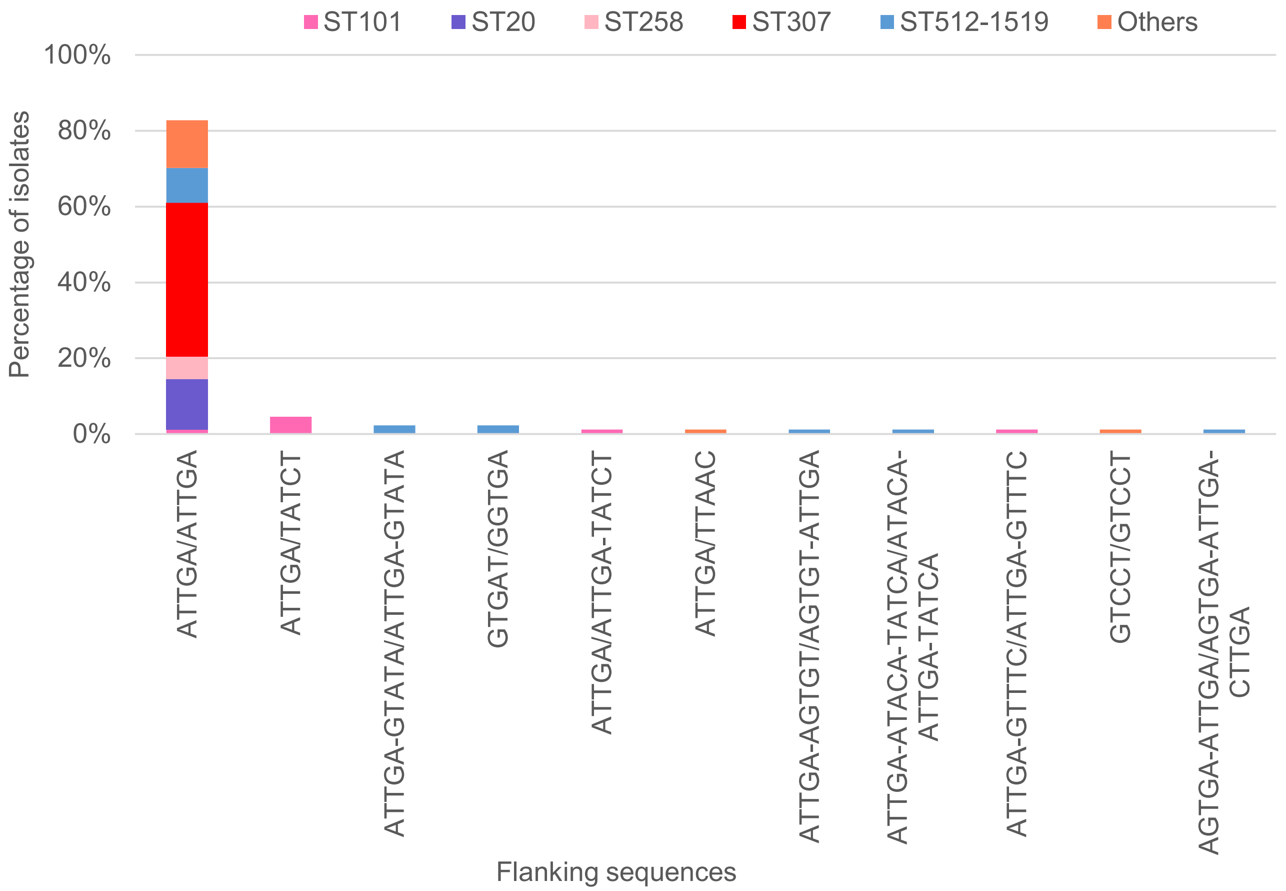

Supplementary Figure 3. **Schematic representation of the Tn*4401*a flanking regions among the different STs.** Graphical representation of the Tn*4401*a flanking regions among the different STs. The presence of more than 2 sequences denotes the presence of multiple transposon copies, integrated in different regions.
