# Supplementary material for: Genomic characterization of Klebsiella pneumoniae carbapenemase-producing Klebsiella pneumoniae (KPC-Kp) strains circulating in three university hospitals in Northern Italy over three years
Source: Antimicrob Resist Infect Control. 2024 Jul 3;13:70. doi: 10.1186/s13756-024-01429-x (PMC11223429; doi:10.1186/s13756-024-01429-x)
Supplement: Supplementary file 7 — Additional file 7: Supplementary Figure 4. Distribution of virulence factors among the 5 prevalent STs. Number of virulence factors carried by strains belonging to the different STs. 1 = only enterobactin, 2 = enterobactin + yersiniabactin, 3= enterobactin + yersiniabactin + aerobactin, 4 = enterobactin + yersianabactin+ aerobactin + rmpA2 [file 13756_2024_1429_MOESM7_ESM.pdf]

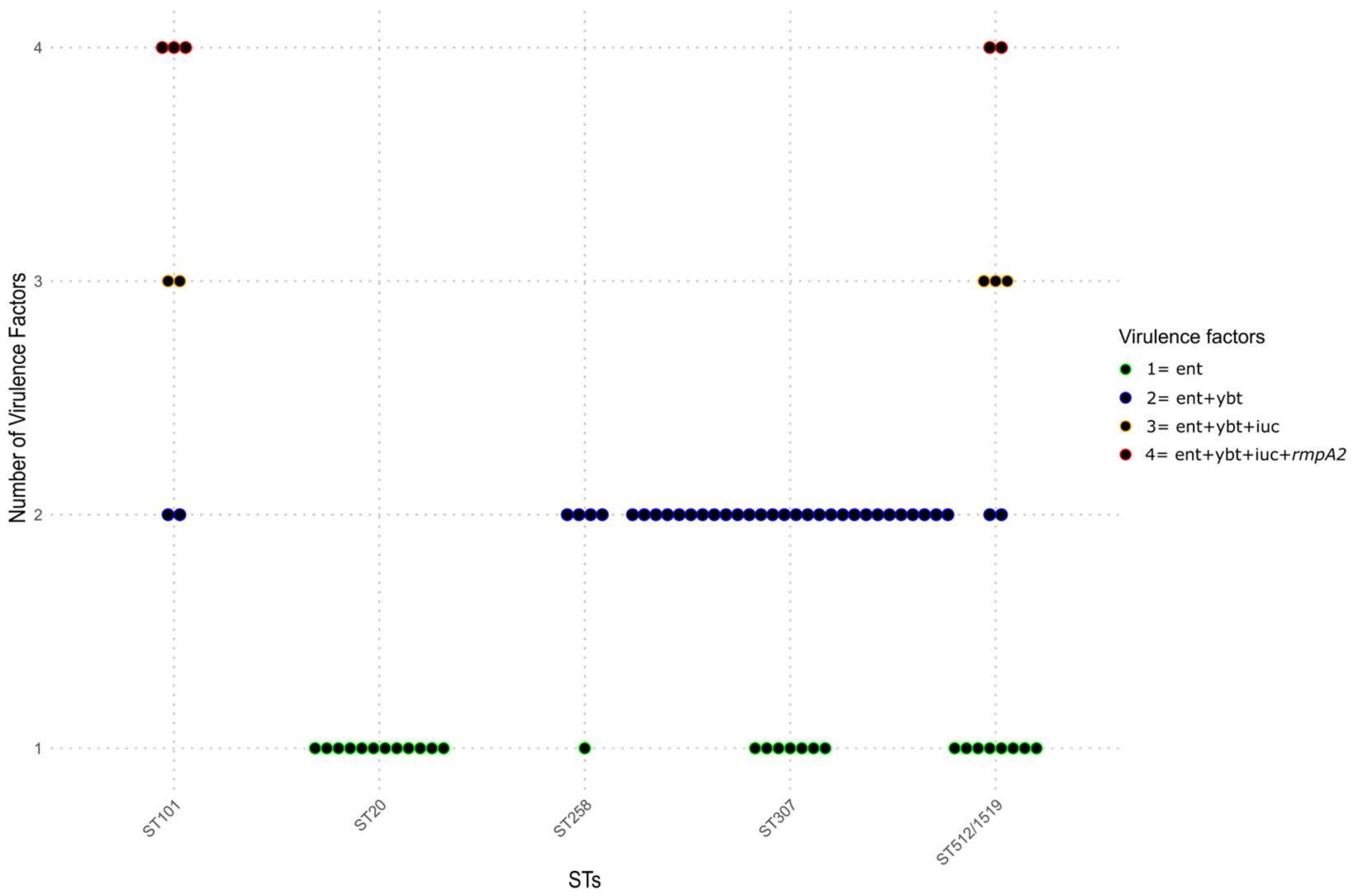

Supplementary Figure 4. **Distribution of virulence factors among the 5 prevalent STs**. Number of virulence factors carried by strains belonging to the different STs. 1 = only enterobactin, 2 = enterobactin + yersiniabactin, 3 = enterobactin + yersiniabactin + aerobactin, 4 = enterobactin + yersianabactin + aerobactin + *rmpA2*.
