# Supplementary material for: Genomic characterization of Klebsiella pneumoniae carbapenemase-producing Klebsiella pneumoniae (KPC-Kp) strains circulating in three university hospitals in Northern Italy over three years
Source: Antimicrob Resist Infect Control. 2024 Jul 3;13:70. doi: 10.1186/s13756-024-01429-x (PMC11223429; doi:10.1186/s13756-024-01429-x)
Supplement: Supplementary file 8 — Additional file 8: Supplementary Table 1. Reference genomes included in phylogenetic analysis. [file 13756_2024_1429_MOESM8_ESM.docx]

**Supplementary table 1**. Reference genomes included in phylogenetic analysis.

| **Strain** | **MLST** | **K locus** | **O locus** | **Carbapenemase gene** | **Isolation source** | **Collection date** | **Isolation country** | **GenBank acc n°** |
| --- | --- | --- | --- | --- | --- | --- | --- | --- |
| KpBO_TO3 | ST512 | KL107 | O2afg | *bla*_KPC-3_ | Blood | 2021-07-01 | Italy | CP090626 |
| 6711.43 | ST258 | KL106 | O2afg | - | Rectal swab | 2018 | Switzerland | CP083007 |
| NRZ-33224 | ST35 | KL110 | O1 | - | Genital swab | 2017-03-30 | Germany | CP084479 |
| RHBSTW-00510 | ST20 | KL24 | O1 | - | Wastewater | 2017 | United Kingdom | CP056432 |
| RIVM_C015657 | ST101 | KL17 | O1 | *bla*_OXA-48_ | N/a | 2017 | Netherlands | CP068994 |
| MCL-2017-2-T0 | ST307 | KL102 | O2afg | - | N/a | 2021-07-01 | Switzerland | CP086467 |
| MRSN752165 | ST147 | KL64 | O2a | *bla*_NDM-1_ | Surveillance | 2019-08-01 | Italy | CP074087 |
| 103-17 | ST1519 | KL107 | O2afg | - | Duodenoscope instr. | 2017 | Italy | CP042310 |
| 51015 | ST15 | KL24 | O1 | *bla*_NDM-1_,  *bla*_OXA-244_ | Rectal swab | 2019-06 | Czech Republic | CP050376 |
| RIVM_C018500 | ST45 | KL24 | O2a | *bla*_OXA-48_ | N/a | 2019 | Netherlands | CP068997 |
| 213293972 | ST37 | KL15 | O4 | *bla*_OXA-48_ | Urine | 2020 | Switzerland | CP083051 |
| 3347689I | ST395 | KL108 | O1 | *bla*_NDM-1_ | Outer malleolus | 2020 | Switzerland | CP071086 |
| LC-1736/18 | ST512 | KL107 | O2afg | *bla*_KPC-3_ | Bronchial aspirate | 2018 | Italy | CP110936 |
| LC-79/19 | ST512 | KL107 | O2afg | *bla*_KPC-3_ | Urine | 2019 | Italy | CP110947 |
| LC-422/19 | ST512 | KL107 | O2afg | *bla*_KPC-3_ | Rectal swab | 2019 | Italy | CP110962 |
| LC-424/19 | ST1519 | KL107 | O2afg | *bla*_KPC-3_ | Urine | 2019 | Italy | CP110967 |
