# Supplementary material for: Genomic characterization of Klebsiella pneumoniae carbapenemase-producing Klebsiella pneumoniae (KPC-Kp) strains circulating in three university hospitals in Northern Italy over three years
Source: Antimicrob Resist Infect Control. 2024 Jul 3;13:70. doi: 10.1186/s13756-024-01429-x (PMC11223429; doi:10.1186/s13756-024-01429-x)
Supplement: Supplementary file 9 — Additional file 9: Supplementary Table 2. Demographic, and clinical findings of general KPC-Kp population and the 95 KPC-Kp population selected for WGS. [file 13756_2024_1429_MOESM9_ESM.docx]

|  | **General KPC-Kp population, N=136** | **Sampled population for WGS, N=94** | **Odds Ratio (Confidence Interval)^§^** | **P-value^§^** |
| --- | --- | --- | --- | --- |
| **Demographics and clinical characteristics** | | | | |
| **Age, years:** | | | | |
| 18-50 | 15 (11.0) | 8 (8.5) | 0.465 (0.157-1.380) | 0.168 |
| 51-65 | 35 (25.7) | 26 (27.7) | 1.402 (0.591-3.328) | 0.444 |
| >65 | 86 (63.3) | 60 (63.8) | 1.086 (0.512-2.303) | 0.830 |
| **Sex, Male** | 96 (70.6) | 65 (69.1) | 0.795 (0.352-1.797) | 0.582 |
| **Infection events** | 43 (31.6) | 35 (37.2) | 2.521 (1.050-6.056) | 0.039 |
| **Isolation date** | | | | |
| January-June 2019 | 15 (11.0) | 13 (13.8) | 2.927 (0.625-13.706) | 0.173 |
| July-December 2019 | 26 (19.1) | 25 (26.6) | 14.855 (1.940-113.766) | 0.009 |
| January-June 2020 | 7 (5.1) | 7 (7.4) | 3.5x10^-8^ (3.5x10^-8^-3.5x10^-8^) | 0.699 |
| July-December 2020 | 16 (11.8) | 10 (10.6) | 0.714 (0.241-2.113) | 0.543 |
| January-June 2021 | 39 (28.7) | 25 (26.6) | 0.725 (0.330-1.593) | 0.423 |
| July-December 2021 | 33 (24.3) | 14 (14.9) | 0.212 (0.092-0.487) | <0.001 |
| **Isolation site** | | | | |
| Rectal swab | 68 (50.0) | 41 (43.6) | 0.430 (0.203-0.911) | 0.028 |
| Urine | 27 (19.9) | 21 (22.4) | 1.726 (0.641-4.651) | 0.280 |
| Blood | 23 (16.9) | 19 (20.2) | 2.407 (0.765-7.575) | 0.133 |
| Others^a^ | 18 (13.2) | 13 (13.8) | 1.188 (0.394-3.576) | 0.760 |
| **Hospital** | | | | |
| P | 88 (64.7) | 60 (63.8) | 0.882 (0.410-1.900) | 0.749 |
| H | 31 (22.8) | 25 (26.6) | 2.174 (0.818-5.780) | 0.120 |
| S | 17 (12.5) | 9 (9.6) | 0.450 (0.160-1.263) | 0.129 |

**Supplementary Table 2.** Demographic, and clinical findings of general KPC-Kp population and the 94 KPC-Kp population selected for WGS**.**

Data are expressed as median (IQR), or N (%). ^§^For comparisons of demographic and clinical findings between general and selected KPC-Kp populations, a Likelihood Ratio Test followed by a multinomial logistic regression model to estimate 95% confidence intervals of odds ratios was used. Two-sided P-values are reported. ^a^Others include: Abdomen-pelvis (n=8), Respiratory sample (n=6), and Other surveillance swabs (n=4)
